# The relationship between polyunsaturated fatty acids and inflammation: evidence from cohort and Mendelian randomization analyses

**DOI:** 10.1093/ije/dyaf065

**Published:** 2025-06-24

**Authors:** Daisy C P Crick, Sarah L Halligan, George Davey Smith, Golam M Khandaker, Hannah J Jones

**Affiliations:** MRC Integrative Epidemiology Unit, Population Health Sciences, Bristol Medical School, University of Bristol, Bristol, United Kingdom; Population Health Sciences, Bristol Medical School, University of Bristol, Bristol, United Kingdom; Institute for Molecular Bioscience, The University of Queensland, Brisbane, Queensland, Australia; Department of Psychology, University of Bath, Bath, United Kingdom; Department of Psychiatry and Mental Health, University of Cape Town, Cape Town, South Africa; Department of Psychiatry, Stellenbosch University, Stellenbosch, South Africa; MRC Integrative Epidemiology Unit, Population Health Sciences, Bristol Medical School, University of Bristol, Bristol, United Kingdom; Population Health Sciences, Bristol Medical School, University of Bristol, Bristol, United Kingdom; MRC Integrative Epidemiology Unit, Population Health Sciences, Bristol Medical School, University of Bristol, Bristol, United Kingdom; Population Health Sciences, Bristol Medical School, University of Bristol, Bristol, United Kingdom; National Institute of Health and Care Research Bristol Biomedical Research Centre, University Hospitals Bristol and Weston NHS Foundation Trust and University of Bristol, Bristol, United Kingdom; Avon and Wiltshire Mental Health Partnership NHS Trust, Bristol, United Kingdom; Centre for Academic Mental Health, Population Health Sciences, Bristol Medical School, University of Bristol, Bristol, United Kingdom; MRC Integrative Epidemiology Unit, Population Health Sciences, Bristol Medical School, University of Bristol, Bristol, United Kingdom; Population Health Sciences, Bristol Medical School, University of Bristol, Bristol, United Kingdom; National Institute of Health and Care Research Bristol Biomedical Research Centre, University Hospitals Bristol and Weston NHS Foundation Trust and University of Bristol, Bristol, United Kingdom; Centre for Academic Mental Health, Population Health Sciences, Bristol Medical School, University of Bristol, Bristol, United Kingdom

**Keywords:** inflammation, polyunsaturated fatty acids, biomarkers, CRP, IL-6, GlycA, ALSPAC, Mendelian randomization, FADS, ELOVL2

## Abstract

**Background:**

Omega-3 (n-3) and omega-6 (n-6) polyunsaturated fatty acids (PUFAs) are thought to have anti- and pro-inflammatory roles, respectively, and influence the risk of various chronic diseases. However, it is unclear whether these associations are causal.

**Methods:**

We examined the associations of dietary polyunsaturated FAs with biomarkers of systemic inflammation: C-reactive protein (CRP), glycoprotein acetyls (GlycA), and interleukin 6 (IL-6) in two cohort datasets—Avon Longitudinal Study of Parents and Children (*N* = 2802) and UK Biobank (*N* = 12 401)—by using multivariable analyses. We investigated causality by using two-sample Mendelian randomization (MR). In addition to the inverse-variance weighted (IVW) method, we used sensitivity analyses to strengthen the causal inference. We conducted multivariable MR (MVMR) to investigate the causal effects of n-3 and n-6 on inflammation, accounting for the low-density lipoprotein (LDL) cholesterol, triglycerides, monounsaturated FAs, and saturated FAs.

**Results:**

Cohort analyses show a positive association between the n-6:n-3 ratio and each biomarker. Total n-3 and n-6 PUFAs were associated with higher GlycA levels [mean difference = 0.33; 95% confidence interval (CI) = 0.29, 0.36, and 0.52; 95% CI = 0.48, 0.55, respectively]. The MR results suggest that total n-3 FAs cause higher circulating CRP (IVW = 0.09; 95% CI = 0.03, 0.16) and GlycA levels (0.12; 95% CI = 0.04, 0.21). The positive association between n-3 FAs and GlycA remained in the MVMR analysis after accounting for LDL cholesterol, triglycerides, monounsaturated FAs, and saturated FAs.

**Conclusion:**

We find no convincing evidence of a simple pro- and anti-inflammatory dichotomy regarding the function of n-6 and n-3 PUFAs. Further research is needed to better understand the mechanisms underlying the effects of PUFAs on specific immune biomarkers.

Key Messages

- Omega-3 (n-3) and omega-6 (n-6) polyunsaturated fatty acids (PUFAs) are thought to have anti- and pro-inflammatory roles, respectively, but it is unclear whether these associations are causal.
- Contrary to current understanding, n-3 FAs are not associated with lower inflammatory marker levels [C-reactive protein (CRP) or glycoprotein acetyls]. We report that both n-3 and n-6 PUFAs are associated with higher GlycA and CRP levels.
- Our findings argue against the presence of a simple pro- and anti-inflammatory dichotomy regarding the function of n-6 and n-3 PUFAs, respectively, and suggest that n-3 supplementation alone may not reduce systemic inflammation.

## Introduction

Polyunsaturated fatty acids (PUFAs) are proposed to influence systemic inflammation [1]. There are two families of PUFAs that are essential for many metabolic processes: omega-3 (n-3) [2, 3] and omega-6 (n-6) [3]. Some PUFAs, like docosahexaenoic acid [DHA; an n-3 PUFA (22:6-n3)] can come from both exogenous sources (such as meat, egg products, and seafood) and endogenous sources (synthesized from their metabolomic precursors through desaturation and elongation reactions) [4]. However, linoleic acid [LA: an n-6 PUFA (18:2-n6)] can only be acquired through dietary consumption [5].

Evidence suggests that n-3 PUFAs have anti-inflammatory effects and subsequently reduce the severity/occurrence of inflammation-related conditions [6, 7] given that cytokines and acute-phase proteins are implicated in the pathophysiology of many non-communicable diseases (NCDs) [8–16]. A meta-analysis of 14 clinical trials (*n* = 135 291) suggested that n-3 supplementation reduced the risk of adverse cardiovascular events/death [17]. However, a randomised control trial (RCT) investigating dietary intake and mortality in 3114 men with angina found that risk of cardiac death was higher among individuals advised to eat oily fish/take fish oil compared with a control group [18]. Additionally, studies have reported that the consumption of n-6 PUFAs had no effect on concentrations of inflammatory biomarkers, despite reports that they were pro-inflammatory [19–22]. However, these studies do not directly assess the impact of FAs on inflammation and the contradictory results raise questions of whether PUFAs ‘causally’ affect inflammation or whether changes to inflammatory biomarker levels are the result of residual confounding.

Both n-3 and n-6 PUFAs are metabolized by the same enzymes and compete for desaturation and elongation. Therefore, n-6 PUFAs act as competitive inhibitors of n-3 PUFAs and thus reduce the amount of end-product n-3 PUFAs that can be synthesized [23]. Therefore, the plasma n-6:n-3 ratio may also impact the concentration of inflammatory makers and subsequent health [24–27]. Research to understand the importance of the n-6:n-3 ratio on levels of inflammation and its impact on the occurrence of NCDs is of public health concern.

We investigated the effect of specific n-3 and n-6 PUFAs, which play a role in key metabolic processes on inflammatory markers, by using (i) traditional observational epidemiological analyses and (ii) Mendelian randomization (MR) [28].

Our exposures were DHA and LA, positioned at opposite ends of the biosynthesis pathways (Supplementary Figure S1). To further interrogate the PUFA–inflammation relationship, we included total n-3 PUFAs, total n-6 PUFAs, and the ratio of total n-6:total n-3 PUFAs. Outcomes included C-reactive protein (CRP) and interleukin-6 (IL-6)—commonly used biomarkers of systemic inflammation and glycoprotein acetyls (GlycA). GlycA are derived from multiple proteins involved in the acute-phase response and consequently is thought to be a more stable marker of inflammation compared with single proteins such as CRP [29–31]. Our aim was to assess how PUFAs affect inflammation by using specific and broad measures.

## Methods

### Observational epidemiological analyses

The primary analysis used the Avon Longitudinal Study of Parents and Children (ALSPAC) birth cohort that recruited a total of 14, 541 pregnant women residing in South-West England [32–34]. For eligibility criteria, see the study flowchart (Supplementary Figure S2). Participants were included if they had exposure (DHA, LA, total n-3, and total n-6) and outcome (CRP, IL-6, and GlycA levels) data available at 24 years.

We used maternal self-reported highest educational qualification and highest occupation of either parent [measures of social economic position (SEP)], maternal and paternal smoking pattern during pregnancy (measures of pregnancy health), participants’ sex, smoking status, alcohol-drinking status, and triglyceride, low-density lipoprotein cholesterol (LDL cholesterol), saturated fatty acid (SFA), and monounsaturated fatty acid (MUFA) levels at 24 years as covariates.

For additional information on ALSPAC, blood sampling, and data collection/processing, see Supplementary Materials.

Linear regression analyses were used to examine the cross-sectional associations of PUFA levels with GlycA, CRP, and IL-6 levels at age 24 years. We ran an unadjusted model; a model adjusted for sex, age, maternal and paternal smoking patterns during pregnancy and SEP (Model 2); a model including all covariates from Model 2 and triglycerides and LDL cholesterol (Model 3); and finally a model that included all covariates from Model 3 and SFAs and MUFAs (Model 4). Due to high correlations between PUFAs, lipids, MUFAs, and SFAs, results from Model 2 were used as our main findings. Results from Models 3 and 4 should be interpreted with multicollinearity in mind (variance inflation factors are presented in Supplementary Figure S3). Details about data cleaning are presented in the Supplementary Materials. Analyses were run by using the whole sample and sex-stratified.

We used multiple imputation (described in detail in the Supplementary Materials) to impute missing exposure, outcome, and covariate data in the eligible sample (*N* = 2802).

We replicated the analysis by using data from UK Biobank (UKB) (*N* = 12 401)—a community-based, prospective study (https://www.ukbiobank.ac.uk) [35]. Despite, the larger sample size, UKB served as replication because it was used in the MR analysis. To strengthen confidence in the results (triangulation), we used different methods in different cohorts [36]. Information about UKB and the replication analysis is presented in the eMethods.

### Two-sample MR analysis

We conducted two-sample MR by using genome-wide significant single-nucleotide polymorphisms (SNPs; *P *< 5.0 ×10^−8^) as instrumental variables (IVs) from European Genome Wide Association Studies (GWAS) of PUFAs [37], GlycA [38], CRP [39], and IL-6 [40]. SNPs were harmonized, aligning the genetic association for exposure and outcome on the effect allele by using the effect allele frequency. See Supplementary Materials for details on instrument selection and the number of remaining instruments.

The inverse-variance weighted [41] method was the primary analysis used to calculate the effect estimates. MR–Egger, weighted median, and weighted mode methods were used as sensitivity analyses. As these methods make different assumptions about instrument validity, consistency across primary and sensitivity analyses increased the confidence that findings were robust. Methods to investigate pleiotropy and heterogeneity are described in the Supplementary Materials.

We replicated the MR analysis by using different outcome datasets and the results were consistent with those of the primary analysis. The GWAS used in this secondary analysis and results are discussed in the Supplementary Materials.

### MR analysis focusing on specific PUFA genes

The *FADS* gene cluster and *ELOVL2* gene encode key desaturase and elongase enzymes, respectively, and are involved in the n-3 and n-6 FA biosynthesis pathways. Therefore, we conducted a complementary and mechanistically informative MR analysis by using only SNPs from within or close to (±500 kb) the *FADS* gene cluster (*FADS1*, *FADS2*, and *FADS3*; chromosome 11:61 560 452–61 659 523) and the *ELOVL2* gene (chromosome 6:10 980 992–11 044 624) from the DHA GWAS and the LA GWAS. SNPs were selected and harmonized by using the same method as in the primary analysis. Details are presented in the Supplementary Materials.

### Multivariable MR analysis

We conducted a multivariable MR (MVMR) analysis [42] (MVMR-1) to estimate the independent direct causal effects of n-3 and n-6 PUFAs on the biomarkers of inflammation. Although SNPs within the FADS region should be reliable instruments of PUFAs due to their proximal relation to PUFA biosynthesis, results may be biased because the SNPs can also be associated with non-fatty acid traits, such as LDL cholesterol and triglycerides [43]. Therefore, we ran two further MVMR analyses: MVMR-2—as in MVMR-1 but also accounting for triglycerides and LDL cholesterol; and MVMR-3—as in MVMR-2 but also accounting for MUFAs and SFAs. Diagrams representing the intertwined metabolic pathway of LDL cholesterol, triglycerides, and FAs, and their effects on inflammation are presented in Supplementary Figure S4.

MVMR analyses are further discussed in the Supplementary Materials.

We used MR-Lap to assess potential bias in effect estimates due to sample overlap, winner’s curse, or weak instruments [44]. Given that SNPs can influence the outcome in distinct ways via discrete biological mechanisms [45], we explored heterogeneity by using MR-Clust [45]. Greater details about MR-Lap and MR-Clust are provided in the Supplementary Materials.

## Results

**Figure 1.**
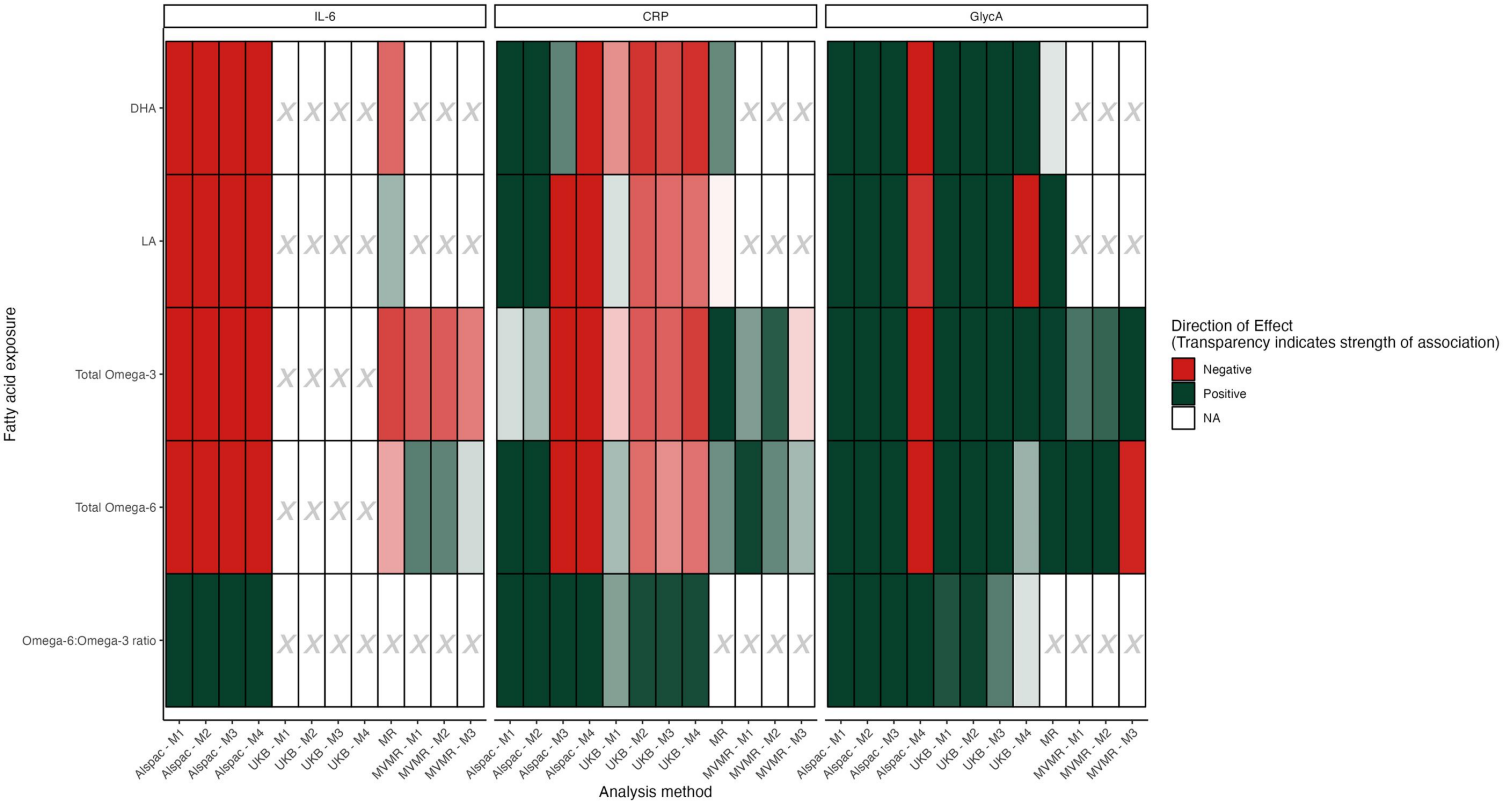
Summary figure of results from the cohort, MR, and MVMR analyses. Green represents a positive association whereas red represents a negative association. Transparency indicates the strength of the association, where bold colours are used for smaller *P*-values and muted colours are used for larger *P*-values. For cohort analyses (ALSPAC and UKB)—M1: no adjustment; M2: adjustment for sex, age, maternal and paternal smoking patterns during pregnancy, and SEP; M3: adjustment for covariates from M2, triglycerides, and LDL cholesterol; M4: covariates included in M3, SFAs, and MUFAs. For MVMR analyses—M1: inclusion of both total n-3 and total n-6 as exposures; M2: inclusion of exposures from M1, triglycerides, and LDL cholesterol; M3: inclusion of exposures from M2, SFAs, and monounsaturated FAs.

Table 1 presents the median/range for PUFA levels and inflammatory biomarker levels in ALSPAC and UKB. Results are discussed in detail in the Supplementary Materials. A summary of all results is presented in Figure 1.

**Table 1.** Median and interquartile range of exposure and outcome data

	ALSPAC		**UKB** ^a^	
	Median (IQR)	Range	Median (IQR)	Range
GlycA (mmol/L)	1.22 (1.12, 1.34)	0.84, 2.25	0.007 (–0.66, 0.69)	–3.91, 4.00
CRP (mg/L)	0.87 (0.39, 2.29)	0.1, 70.05	1.33 (0.66, 2.73)	0.08, 77.32
IL-6 (NPX log2)	3.32 (2.96, 3.85)	1.82, 9.86	–	–
DHA	0.11 (0.09, 0.13)	0.04, 0.32	0.004 (–0.68, 0.66)	–3.75, 3.89
LA	2.31 (1.98, 2.68)	0.89, 4.91	0.0002 (–0.67, 0.67)	–3.59, 4.20
Total n-3 PUFAs	0.29 (0.24, 0.34)	0.07, 0.80	0.01 (–0.67, 0.67)	–3.73, 3.60
Total n-6 PUFAs	2.85 (2.48, 3.28)	1.23, 5.82	–0.001 (–0.66, 0.67)	–3.66, 4.06

IQR, interquartile range; NPX, normalized protein expression value.

a UKB data are presented as inverse normal transformed with exception of CRP due to data availability.

### Results for primary analyses (Model 2) using ALSPAC cohort data

Results for all models are presented in Figure 1 and Table 2.

**Table 2.** Associations of PUFA and inflammatory biomarkers using imputed ALSPAC data (*n *= 2802)

	Model 1 (unadjusted)		**Model 2** ^a^		**Model 3** ^b^		**Model 4** ^c^	
Outcome	Mean difference per SD increase in exposure (se)	95% CI	Mean difference per SD increase in exposure (se)	95% CI	Mean difference per SD increase in exposure (se)	95% CI	Mean difference per SD increase in exposure (se)	95% CI
**Exposure: DHA at age 24 y**								
IL-6 at 24 y	–0.13 (0.02)	–0.16, –0.09	–0.13 (0.02)	–0.17, –0.09	–0.19 (0.02)	–0.23, –0.15	–0.20 (0.03)	–0.25, –0.15
CRP at 24 y	0.12 (0.02)	0.08, 0.16	0.09 (0.02)	0.05, 0.13	0.02 (0.02)	–0.02, 0.06	–0.06 (0.03)	–0.11, –0.01
GlycA at 24 y	0.28 (0.02)	0.25, 0.32	0.31 (0.02)	0.27, 0.35	0.12 (0.02)	0.09, 0.15	–0.09 (0.02)	–0.12, –0.05
**Exposure: LA at age 24 y**								
IL-6 at 24 y	–0.08 (0.02)	–0.12, –0.04	–0.07 (0.02)	–0.11, –0.04	–0.25 (0.02)	–0.30, –0.21	–0.38 (0.04)	–0.45, –0.31
CRP at 24 y	0.09 (0.02)	0.05, 0.12	0.08 (0.02)	0.04, 0.11	–0.10 (0.02)	–0.14, –0.05	–0.45 (0.04)	–0.52, –0.38
GlycA at 24 y	0.47 (0.02)	0.44, 0.51	0.48 (0.02)	0.45, 0.51	0.26 (0.02)	0.22, 0.29	–0.04 (0.03)	–0.10, 0.01
**Exposure: Total n-3 at age 24 y**								
IL-6 at 24 y	–0.15 (0.02)	–0.19, –0.11	–0.14 (0.02)	–0.18, –0.10	–0.27 (0.02)	–0.31, –0.23	–0.33 (0.03)	–0.38, –0.27
CRP at 24 y	0.004 (0.02)	–0.03, 0.04	0.01 (0.02)	–0.03, 0.04	–0.13 (0.02)	–0.17, –0.09	–0.32 (0.03)	–0.37, –0.26
GlycA at 24 y	0.31 (0.02)	0.28, 0.35	0.32 (0.02)	0.29, 0.36	0.07 (0.02)	0.03, 0.10	–0.19 (0.02)	–0.23, –0.16
**Exposure: Total n-6 at age 24 y**								
IL-6 at 24 y	–0.06 (0.02)	–0.09, –0.02	–0.06 (0.02)	–0.09, –0.02	–0.25 (0.03)	–0.30, –0.20	–0.58 (0.05)	–0.68, –0.48
CRP at 24 y	0.15 (0.02)	0.11, 0.18	0.13 (0.02)	0.09, 0.16	–0.02 (0.03)	–0.07, 0.03	–0.57 (0.05)	–0.67, –0.47
GlycA at 24 y	0.51 (0.02)	0.47, 0.54	0.51 (0.02)	0.48, 0.55	0.31 (0.02)	0.27, 0.35	–0.13 (0.04)	–0.20, –0.06
**Exposure: Total-6: total n-3 at age 24 y**								
IL-6 at 24 y	0.20 (0.02)	0.16, 0.23	0.18 (0.02)	0.15, 0.22	0.18 (0.02)	0.15, 0.22	0.17 (0.02)	0.13, 0.21
CRP at 24 y	0.21 (0.02)	0.17, 0.24	0.17 (0.02)	0.14, 0.21	0.17 (0.02)	0.13, 0.20	0.16 (0.02)	0.12, 0.19
GlycA at 24 y	0.18 (0.02)	0.14, 0.22	0.16 (0.02)	0.13, 0.20	0.15 (0.01)	0.12, 0.17	0.14 (0.01)	0.11, 0.16

SD, standard deviation; se, standard error; CI, confidence interval.

a Model 2: Estimates adjusted for household social class at birth, maternal highest education qualification at birth, maternal and paternal smoking status during pregnancy, offspring sex at birth, type of drinker at age 24 y, type of smoker at 24 y, and at age in months at 24-y clinic.

b Model 3: As Model 2 plus additional adjustment for LDL cholesterol (mmol/l) and triglycerides (mmol/L) at the 24-y clinic.

c Model 4: As Model 3 plus additional adjustment for SFAs (mmol/L) and monounsaturated fatty acids (mmol/L) at the 24-y clinic.

DHA and LA were associated with higher CRP and GlycA levels, but lower IL-6 levels after adjusting for Model 2 confounders.

Total n-3 and n-6 PUFAs were associated with higher GlycA levels, but lower IL-6 levels after adjusting for Model 2 confounders. Total n-6 was associated with higher CRP levels and there was no strong evidence of an association between total n-3 PUFAs and CRP after adjusting for Model 2 confounders. The total n-6:n-3 ratio was associated with higher levels of all three biomarkers after adjusting for Model 2 confounders.

In the sex-stratified analyses, the n-6:n-3 ratio was associated with higher levels of all three biomarkers in both sexes (Figure 3 and Supplementary Table S1). PUFAs were associated with higher GlycA in both sexes. The effects of all PUFAs on CRP and IL-6 levels attenuated to the null in males. In females, findings mirrored the results from the whole cohort.

**Figure 2.**
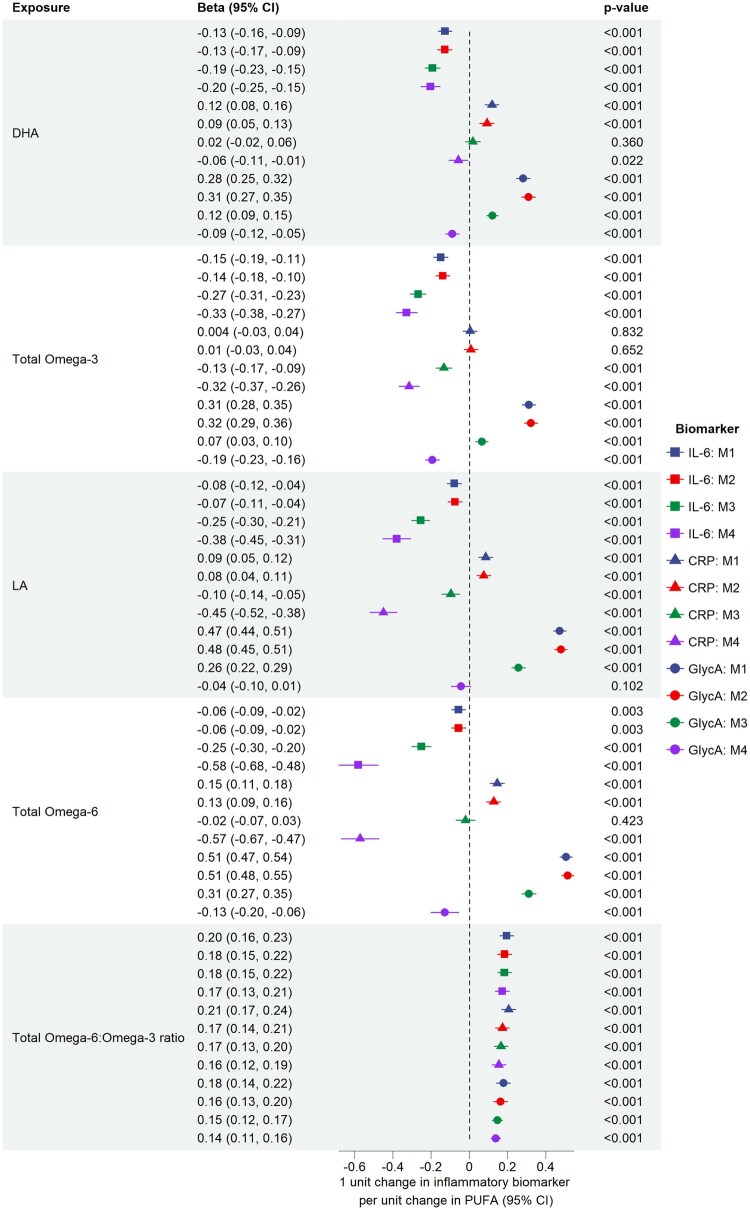
Association between fatty acids and inflammatory biomarkers using ALSPAC data at age 24 y. M1: no adjustment; M2: adjustment for sex, age, maternal and paternal smoking patterns during pregnancy, and SEP; M3: adjustment for covariates from M2, triglycerides, and LDL cholesterol; M4: adjustment for covariates included in M3 and SFAs and monounsaturated fatty acids.

Results when including all listed covariates are presented in the Supplementary Materials and Figure 2. In all analyses, the inclusion of triglycerides and LDL cholesterol (Model 3) and SFAs and MUFAs (Model 4) shifted the effect size towards the negative. With the exception of the n-6:n-3 ratio, the Model 4 shift of effect changed the direction of the effect of all PUFA measures on the CRP and GlycA levels (Figure 2). Given that MUFAs and PUFAs are highly correlated, these results should be interpreted with multicollinearity in mind.

**Figure 3.**
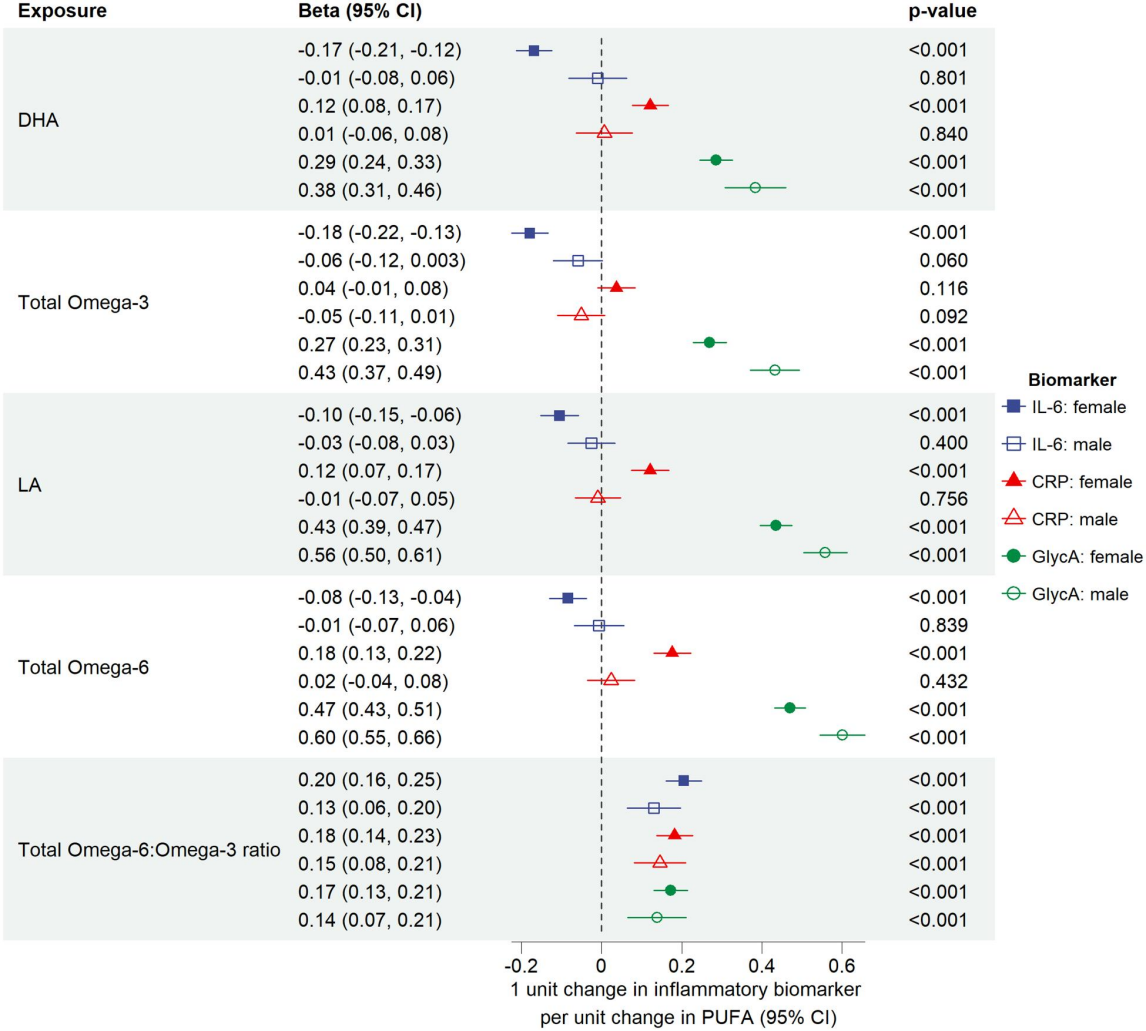
Association between fatty acids and biomarkers of inflammation using ALSPAC data at age 24 y stratified by sex at birth. Analyses presented were adjusted for household social class, maternal highest education qualification, maternal and paternal smoking status during pregnancy, and participant age and smoking and drinking status when attending the ALSPAC 24-y clinic.

Results when using UKB cohort data are presented in the Supplementary Materials. Results for Model 2 are consistent with findings when using ALSPAC data; however, when including the additional covariates (triglycerides, LDL cholesterol, SFAs, and MUFAs), the findings diverged.

### Results from MR analyses

Tests investigating instrument validity indicated that instruments were unlikely to be subject to weak instrument bias (see Supplementary Materials). MR results are presented in Supplementary Figure S5.

We observed no strong evidence of an effect of DHA levels on CRP, GlycA, or IL-6. These results were largely consistent across sensitivity analyses.

We observed no strong evidence of an effect of LA levels on CRP or IL-6. However, estimates suggest that higher LA levels cause higher GlycA levels, which is consistent with results from the cohort analyses. Other than in the analysis investigating the effect of LA levels on CRP, results were consistent across the MR sensitivity methods.

Higher total n-3 PUFA levels were associated with higher GlycA levels (consistently with the cohort analyses). There was also evidence to suggest that higher total n-3 PUFA levels were associated with higher CRP and this was replicated in the secondary MR analysis. We found no effect of total n-3 on IL-6. Results attenuated to the null for sensitivity analyses of total n-3 PUFA levels on GlycA.

We observed no strong evidence of an effect of total n-6 PUFA levels on CRP or IL-6. However, estimates did suggest that higher n-6 PUFA levels cause higher GlycA levels (consistently with the cohort analyses). The MR effect estimates were fairly consistent with the main MR analysis.

Pleiotropy and heterogeneity tests are discussed in the Supplementary Materials, along with the replication analysis. In short, there was no strong evidence of pleiotropy, but there was evidence of heterogeneity. Replication was fairly consistent with the main MR analysis.

### Results from the MVMR analyses

**Figure 4.**
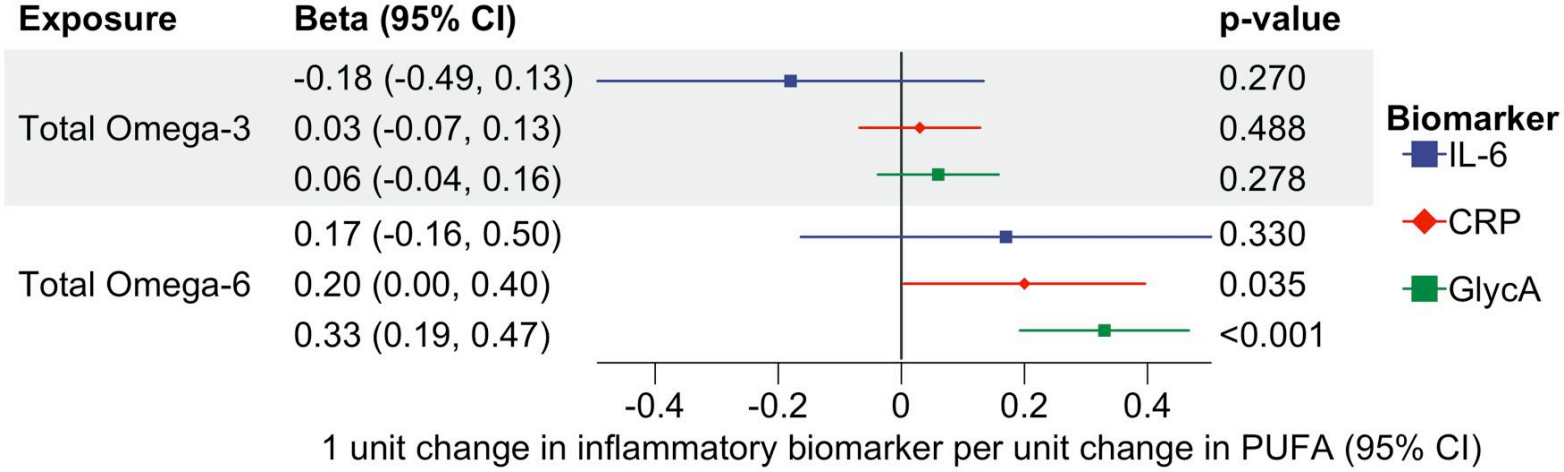
Multivariable MR analysis investigating the direct effect of n-3 and n-6 on three biomarkers of inflammation (CRP, IL-6, and GlycA) independently of each other by accounting for their mutual relationships.

**Figure 5.**
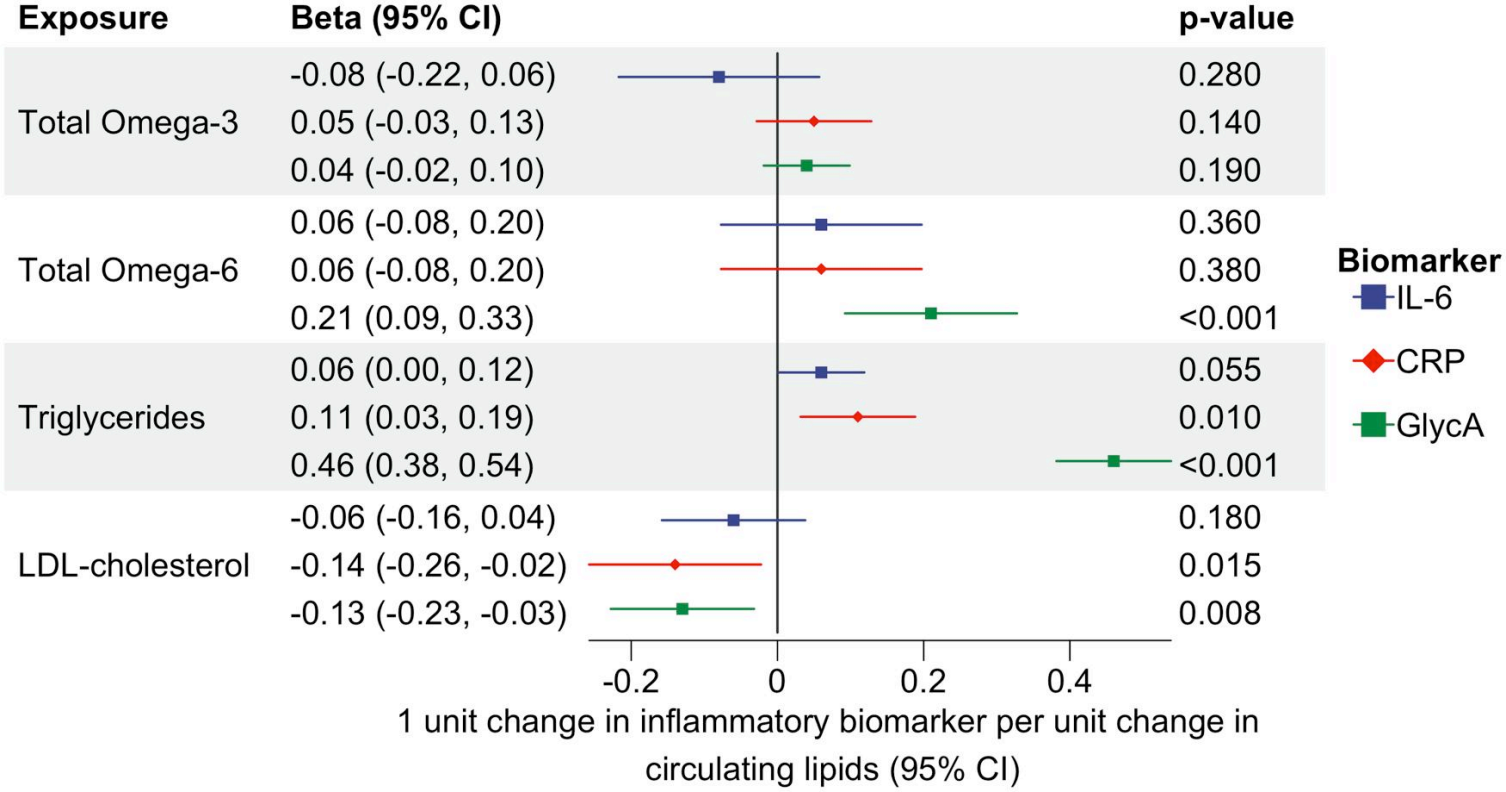
Multivariable MR analysis estimating the effects of n-3, n-6, triglycerides, and LDL cholesterol on three biomarkers of inflammation (CRP, IL-6, and GlycA) independently of each other by accounting for their mutual relationships. 95% CI, 95% confidence interval.

**Figure 6.**
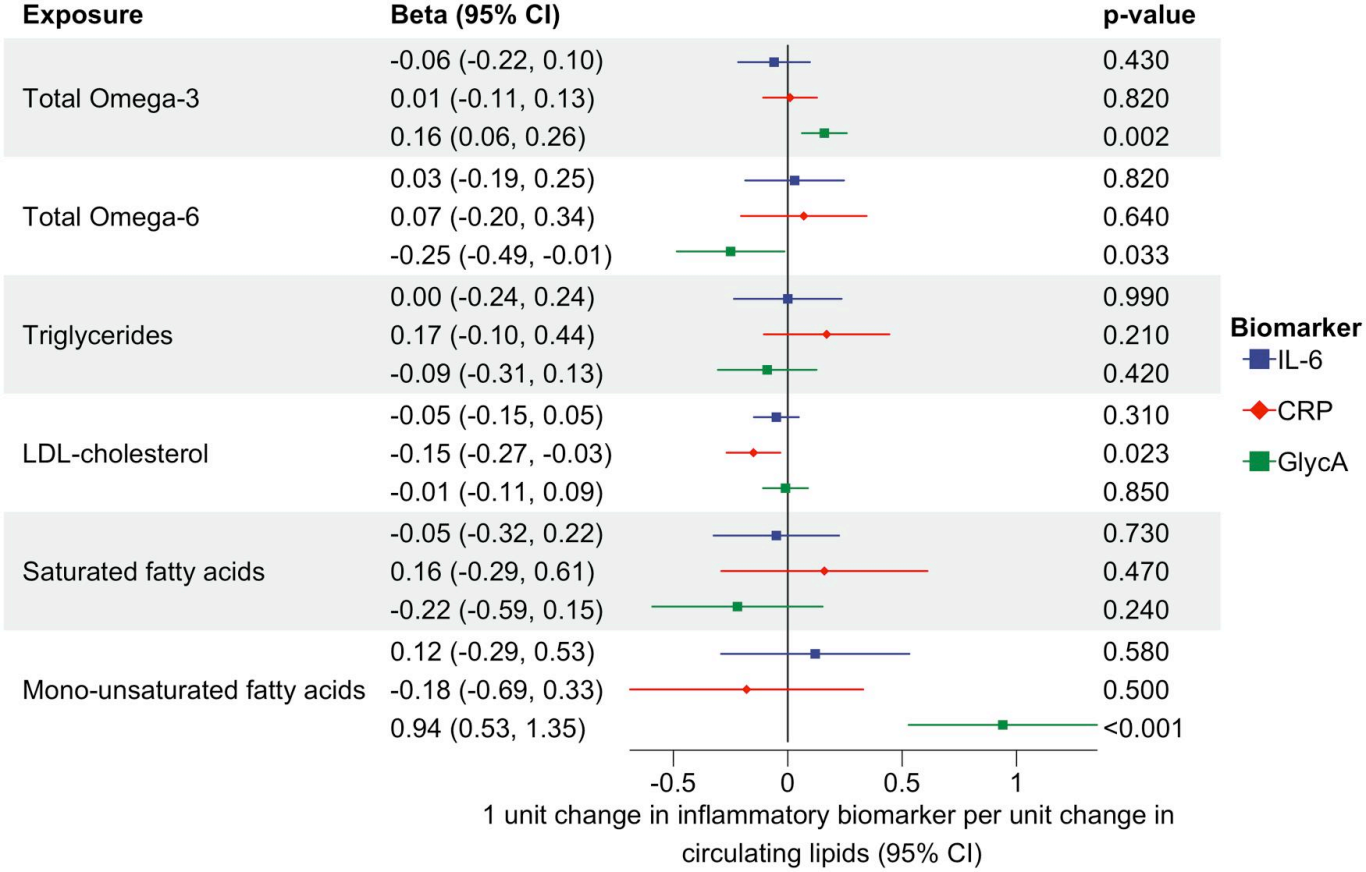
Multivariable MR analysis estimating the direct effects of n-3, n-6, triglycerides, LDL cholesterol, SFAs, and monounsaturated fatty acids on CRP, IL-6, and GlycA independently of each other by accounting for their mutual relationships. 95% CI, 95% confidence interval.

MVMR analyses suggested that total n-6 PUFA levels independently increased both GlycA and CRP levels, but not IL-6 levels, after controlling for the effect of n-3 PUFAs. There was no direct independent effect of total n-3 PUFA levels on GlycA, CRP, or IL-6 after controlling for the effect of n-6 PUFAs (Figure 4), suggesting that pleiotropy may have biased the univariable MR. After further accounting for the effect of triglycerides and LDL cholesterol, we found that total n-6 PUFA levels independently increased the levels of GlycA, but not CRP levels. There was no independent effect of total n-3 PUFAs on any biomarker (Figure 5). A further description of the results, including the third MVMR, in which triglycerides, LDL cholesterol, SFAs, and MUFAs (Figure 6) were also included, is presented in the Supplementary Materials along with results from MR-Lap.

### Results for MR analyses focusing on specific PUFA genes

MR analyses using SNPs from the *FADS* gene region suggested that DHA increased CRP levels, but that LA decreased CRP levels. *FADS* instrumented DHA and LA had no effect on GlycA or, where testable, IL-6. There was no evidence of a causal effect of any SNPs from the *ELOVL2* gene on GlycA, CRP, or IL-6. See the Supplementary Materials for further information.

### Results from the MR-Clust analysis

As SNPs can influence outcomes in distinct ways and associations between PUFAs and GlycA were consistent across the cohort and MR analyses, we used MR-Clust [45] to identify SNP clusters driving heterogeneity. For LA, total n-3, and total n-6 PUFAs, all clusters showed positive associations with GlycA, aligning with the main result (Supplementary Figures S6–S8). For DHA, two clusters were positively associated with GlycA whereas one showed a negative association (Supplementary Figure S9). All three showed gene enrichment in lipid pathways/lipid-related GWAS. No cluster-related differentially expressed gene sets met statistical significance for tissue enrichment (Supplementary Tables S2–S6 and Supplementary Figure S10). Detailed results are presented in the Supplementary Materials.

## Discussion

By combining cohort and genetic analyses, we provide insight into the relationships between PUFAs and systemic inflammation. Using multivariable linear regression, we found evidence that DHA, LA, total n-3, and total n-6 increased levels of GlycA (in ALSPAC and UKB). However, results between cohorts were less consistent for the effect of FAs on CRP. When using the ALSPAC cohort, we found a consistent positive association between the n-6:n-3 ratio and all three biomarkers, although this was not replicated in UKB.

Subsequent genetic analyses shed light on the complex nature of these associations. First, consistently with the cohort analyses, LA, total n-3, and total n-6 potentially causally increased the levels of circulating GlycA, which suggests a pro-inflammatory effect of these FAs. Further, as with GlycA, the MR results show that total n-3 PUFAs causally increased the circulating CRP levels. This contrasts with the presumed anti-inflammatory effect of n-3 PUFAs. We also replicated this finding by using secondary, independent GWAS data, which increases the confidence in this result.

Our MVMR analyses highlight the potential importance of total n-6 FAs above total n-3 FAs regarding their effect on systemic inflammation. We found, consistent with the cohort and univariable MR analysis, that higher total n-6 FAs increased GlycA levels after accounting for the effect of n-3 FA levels, triglycerides, and LDL cholesterol. Therefore, as suggested by previous literature, n-6 FAs may increase levels of inflammation. In contrast, total n-3 FAs had no effect on GlycA or CRP after accounting for total n-6 FAs, triglycerides, and LDL cholesterol. This brings into question the use of n-3 supplementation as a method to reduce systemic inflammation. Despite this, biomarkers measure different aspects of inflammation (acute versus low-grade chronic inflammation) and the PUFAs affect them differentially. Therefore, by only using three biomarkers of inflammation, we may have missed important effects that PUFAs have on inflammation. Research using additional biomarkers or inflammatory networks is needed to better understand the relationship between PUFA consumption and systemic inflammation.

Results from our MR analyses using genetic variants within/near the *FADS* gene cluster differed from those of the main analysis, indicating that the original results may have been biased by pleiotropy, rather than reflecting the true effect of FA biosynthesis. Even when using genetic variants that fall within the *FADS* gene region, which is key for FA metabolism, the instruments may lack specificity because they are also enriched in genes involved in actions such as lipoprotein metabolism [43]. We aimed to mitigate this problem through the inclusion of lipoproteins in an MVMR, although they too may be impacted by horizontal pleiotropy.

A major strength of our study is that we investigated the association between PUFAs and levels of CRP, IL-6, and GlycA by using two different methods, replicated the methods by using different cohorts, and ran further sensitivity analyses to address potential biases that arise in epidemiological research.

Despite this, we recognize several limitations. Using cross-sectional analyses means that we could not determine the temporal or causal relationship between the exposure and the outcome. However, it does provide useful context and evidence for associations between PUFA levels and biomarkers of inflammation, including GlycA, which is novel.

DHA and LA appear at different ends of FA biosynthesis pathways (DHA is a product of n-3 desaturation/elongation reactions whereas LA is a n-6 precursor), creating difficulties with direct comparisons. Additionally, the nuclear magnetic resonance (NMR) platform did not measure all FAs, meaning that key inflammatory metabolites may have been missed. Although our results suggest that total n-6 increases inflammation, the limited NMR coverage and pleiotropic nature of FA genetic instruments prevent us from confidently identifying the specific classes of FAs that were driving these associations. Conditioning on total fatty acid levels could introduce collider bias and therefore we did not use FAs reported as a proportion of total FAs. Subsequently, the relative abundance of each FA may have been obscured and it is harder to interpret the significance of individual FAs. Further, in some cases, reporting FAs individually may not have provided enough context regarding the overall composition of the individual’s blood, such as the overall lipid profile. Despite this, reporting individual FAs was informative, as this is the first study on their causal link with inflammation.

Another limitation is that inflammation is a latent trait that is inferred through various markers. Circulatory protein levels may not fully capture its biological complexity. Although we failed to observe anti-inflammatory properties, our findings do not rule out anti-inflammatory effects of n-3 PUFAs and work is needed to understand this multifaceted relationship. However, given that the focus of this work was to examine the relationship between PUFAs and systemic inflammation, CRP, IL-6, and GlycA are appropriate, as they reflect broad inflammatory activity.

Lastly, although the *FADS* gene cluster encodes enzymes that are fundamental to PUFA biosynthesis, suggesting that IVs within this region are more likely to satisfy MR assumptions, *FADS* variants have been shown to be highly pleiotropic [46] and do not differentiate n-3 or n-6 effects. As such, our gene-based analyses may have captured pathways to inflammation through factors other than PUFAs, thus violating the exclusion–restriction assumption [28].

Given the popularity of n-3 PUFAs supplements [47–49], our results are of public health importance. Replication could shift messaging toward reducing total n-6 PUFA consumption and promoting a healthy, balanced n-6:n-3 ratio to lower inflammation. However, we acknowledge that n-3 PUFAs have benefits beyond their posited anti-inflammatory effects, e.g. hypotriglyceridaemic properties [50], and that some anti-inflammatory effects may not have been captured by our biomarkers. A better understanding of n-6 and n-3 interactions is needed before policy change.

## Ethics approval

Ethical approval for the study was obtained from the ALSPAC Ethics and Law Committee and the Local Research Ethics Committee and can be found here at http://www.bristol.ac.uk/alspac/researchers/research-ethics/. Informed consent for the use of data collected via questionnaires and clinics was obtained from participants following the recommendations of the ALSPAC Ethics and Law Committee at the time. Consent for biological samples has been collected in accordance with the Human Tissue Act (2004). UKB has approval from the North West Multi-centre Research Ethics Committee as a Research Tissue Bank. Code for data management and statistical analysis has been made available in daisycrick (github.com). Cohort analyses were performed by using STATA version 17.0. All other analyses were performed by using R Software version 4.1.0.

## Supplementary Material

dyaf065_Supplementary_Data

## Data Availability

Data needed to evaluate the conclusions presented in this paper are provided in the manuscript and/or the Supplementary Material. Additionally, ALSPAC data can be requested from the ALSPAC executive committee and reasonable requests from bona fide researchers. GWAS data is publicly available by using the OpenGWAS website (https://gwas.mrcieu.ac.uk). Part of this research was conducted by using data from UKB project ID: 81499 and project: 30418, a major biomedical database, and can be provided by UKB (http://www.ukbiobank.ac.uk/).
